# Genomic epidemiology of the primary methicillin-resistant *Staphylococcus aureus* clones causing invasive infections in Paraguayan children

**DOI:** 10.1128/spectrum.03012-23

**Published:** 2024-02-28

**Authors:** Rosa Guillén, Claudia Salinas, Alejandro Mendoza-Álvarez, Luis A. Rubio Rodríguez, Ana Díaz-de Usera, José M. Lorenzo-Salazar, Rafaela González-Montelongo, Carlos Flores, Fátima Rodríguez

**Affiliations:** 1 Microbiology Department, Instituto de Investigaciones en Ciencias de la Salud, Universidad Nacional de Asunción (IICS-UNA), San Lorenzo, Paraguay; 2 Research Unit, Hospital Universitario Nuestra Señora de Candelaria, Santa Cruz de Tenerife, Spain; 3 Genomics Division, Instituto Tecnológico y de Energías Renovables (ITER), Santa Cruz de Tenerife, Spain; 4 Facultad de Ciencias de la Salud, Universidad Fernando Pessoa Canarias, Las Palmas de Gran Canaria, Spain; 5 CIBER de Enfermedades Respiratorias (CIBERES), Instituto de Salud Carlos III, Madrid, Spain; Instituto de Higiene, Montevideo, Uruguay

**Keywords:** methicillin-resistant *Staphylococcus aureus*, genomic epidemiology, invasive infection, Paraguayan children

## Abstract

Methicillin-resistant *Staphylococcus aureus* (MRSA) is one of the major human pathogens. It could carry numerous resistance genes and virulence factors in its genome, some of which are related to the severity of the infection. An observational, descriptive, cross-sectional study was designed to molecularly analyze MRSA isolates that cause invasive infections in Paraguayan children from 2009 to 2013. Ten representative MRSA isolates of the main clonal complex identified were analyzed with short-read paired-end sequencing and assessed for the virulome, resistome, and phylogenetic relationships. All the genetically linked MRSA isolates were recovered from diverse clinical sources, patients, and hospitals at broad gap periods. The pan-genomic analysis of these clones revealed three major and different clonal complexes (CC30, CC5, and CC8), each composed of clones closely related to each other. The CC30 genomes prove to be a successful clone, strongly installed and disseminated throughout our country, and closely related to other CC30 public genomes from the region and the world. The CC5 shows the highest genetic variability, and the CC8 carried the complete arginine catabolic mobile element (ACME), closely related to the USA300-NAE-ACME+, identified as the major cause of CA-MRSA infections in North America. Multiple virulence and resistance genes were identified for the first time in this study, highlighting the complex virulence profiles of MRSA circulating in the country. This study opens a wide range of new possibilities for future projects and trials to improve the existing knowledge on the epidemiology of MRSA circulating in Paraguay.

**IMPORTANCE:**

The increasing prevalence of methicillin-resistant *Staphylococcus aureus* (MRSA) is a public health problem worldwide. The most frequent MRSA clones identified in Paraguay in previous studies (including community and hospital acquired) were the Pediatric (CC5-ST5-IV), the Cordobes-Chilean (CC5-ST5-I), the SouthWest Pacific (CC30-ST30-IV), and the Brazilian (CC8-ST239-III) clones. In this study, the pan-genomic analysis of the most representative MRSA clones circulating in invasive infection in Paraguayan children over the years 2009–2013, such as the CC30-ST30-IV, CC5-ST5-IV, and CC8-ST8-IV, was carried out to evaluate their genetic diversity, their repertoire of virulence factors, and antimicrobial resistance determinants. This revealed multiple virulence and resistance genes, highlighting the complex virulence profiles of MRSA circulating in Paraguay. Our work is the first genomic study of MRSA in Paraguay and will contribute to the development of genomic surveillance in the region and our understanding of the global epidemiology of this pathogen.

## INTRODUCTION

Methicillin-resistant *Staphylococcus aureus* (MRSA), a major human pathogen, can produce many types of infections, such as food poisoning, necrotizing pneumonia, endocarditis, or septicemia. Initially, MRSA was associated with hospital-acquired infections. However, there has been a considerable increase in cases of healthy individuals who were infected in the community (1, 2). These infections are particularly problematic due to their associated morbidity, length of hospital stay, and mortality (3). MRSA could carry numerous virulence factors, some of which explain the severity of the infections it causes, making it a “superbug” (4).

Nowadays, the complete genome of a microorganism, including the full set of resistance and virulence genes (resistome and virulome, respectively), can be simultaneously characterized by leveraging next-generation sequencing (5). In fact, whole-genome sequencing (WGS) is instrumental in tracking the source and propagation of important MRSA clones. Furthermore, WGS is very useful to rapidly infer the emergence of resistance genotypes in the clinical setting to target widely available optimal therapeutic approaches, especially during invasive infection treatments (6).

Severe infections caused by methicillin-resistant strains acquired in the community (CA-MRSA) in the United States at the beginning of the century were reported. This strain is called USA300 and quickly dispersed geographically, displacing less virulent strains, and becoming the leading cause of skin and soft tissue infections in this country (7–9). The most frequent MRSA clones (including community and hospital-acquired) in South America are the Pediatric (CC5-ST5-IV), the Cordobes-Chilean (CC5-ST5-I), the SouthWest Pacific (CC30-ST30-IV), the Brazilian (CC8-ST239-III), and New York/Japan (CC5-ST5-II) clones (10). In Argentina, the main CA-MRSA clone related to invasive infections in the last decade has been CC30-ST30-IV-t019 PVL+, which became predominant, replacing the previously described CC5-ST5-IV-t311 PVL+ (11–13). In Paraguay, the prevalence of CA-MRSA CC30-ST30-IV clone is the leading cause of *S. aureus* infections both at the regional level and in the pediatric population (11, 14, 15). The objective of this study was the deep characterization of the genomic features of MRSA isolates causing invasive infections in Paraguayan children.

## MATERIALS AND METHODS

### Bacterial strains

An observational, descriptive, and cross-sectional study was designed to analyze representative MRSA isolates of the main clones identified between 2009 and 2013, from the Microbiology Department of the Health Sciences Research Institute, UNA, Paraguay Biobank (maintained at −80°C in BHI + 20% glycerol). Isolates were initially recovered from invasive infections of children under 16 years old attending any of the four reference hospitals from Asunción and the Central Department of Paraguay that collaborated in this study. Invasive infection was defined as a localized or systemic inflammatory response to the presence of *S. aureus* at otherwise sterile anatomical sites (16). Identification data, epidemiology files, and records of antimicrobial susceptibility were extracted from the epidemiological records of the isolates. Phenotypic identification of the isolates and the antimicrobial susceptibility tests were carried out following the criteria recommended by the Clinical and Laboratory Standards Institute from 2009 to 2013, according to the strain collection date or by automated systems using Vitek2 (BioMérieux, La Balme, France) following to the manufacturer’s instructions. Susceptibility to vancomycin was determined by E-test for all isolates.

These strains were sub-cultured in Tryptic Soy Agar (Difco, Le Pont de Claix, France) medium from primary cultures and incubated for 24 h at 35°C under 5% CO_2_ for further molecular characterization.

### Genotyping and DNA extraction

Total bacterial DNA from MRSA samples (*N* = 39) was extracted from pure MRSA cultures using the Wizard Genomic DNA Purification kit (Wizard Genomic, Promega, Madison, USA) following the manufacturer’s instructions. MRSA was molecularly typed by spa typing (17) and multi-locus sequence typing (MLST) (18). Detection of *mecA* and Panton-Valentine leukocidin (PVL)-coding genes and PFGE (pulse field gel electrophoresis) were carried out as described previously (14, 19). The characterization by multi-locus variable analysis (MLVA) was carried out by a multiplex PCR as described elsewhere (20). The *staphylococcal cassette chromosome mec* (*SCCmec*) element was typed using Kondo’s typing system (21).

Based on the data produced by these molecular analyses (Table S1) and due to budget limitations, we carefully selected the 10 most representative isolates of the main MRSA clones causing invasive infections in Paraguayan children that circulated in the studied period for whole-genome sequencing.

### Library preparation and whole-genome sequencing

DNA samples for WGS were purified and concentrated for library preparation with DNA Clean & Concentrator (Zymo Research, CA, USA), following the manufacturer’s recommendations. Dual index libraries were processed with Nextera XT DNA Library Preparation Kit (Illumina Inc., CA, USA) following the manufacturer’s recommendations with manual library normalization. Paired-end sequencing was performed on a MiSeq Sequencing System (Illumina Inc., CA, USA) with 300 base reads to a theoretical sequencing throughput of 3 Mb/library (minimal expected coverage of 100×). The library concentration was loaded at 10 pM, and 5% of PhiX Control V3 (Illumina Inc., CA, USA) was used as the internal control. Sequencing was conducted in the Genomic Division of the Instituto Tecnológico y de Energías Renovables (ITER, Tenerife, Spain).

### Bioinformatic analysis

BCL files were converted to demultiplexed FASTQ files using bcl2fastq v2.19 tool. Quality control was performed with FastQC v0.73 (22) to assess sequencing quality, read length, and the total number of reads. Taxonomic correspondence (species identification) was obtained with Kraken v2.1.1 (23). Then, the reads were subjected to a trimming process to improve their quality using the Trimmomatic software v0.38.1 (24). Subsequently, *de novo* genome assembly was carried out with Unicycler v0.4.8.0 (25) and with Quast Genome assembly Quality v5.0.2 (26, 27) to assess the quality of the assembly. A summary report was obtained with assembly metrics such as total genome size, total number of contigs, largest contig size, and contig with size greater than 1 kb, *N*50, and GC content. Finally, the assemblies were processed with Prokka (Prokaryotic Genome Annotation) v1.14.6 for bacterial annotation (28). All the analyses were conducted on the TeideHPC Supercomputing facility (http://teidehpc.iter.es).

Additional characterization of the isolates was carried out using the assemblies in combination with the software MLST (PubMLST database) v2.19.0 (29, 30) for detecting the sequence type, and the Center for Genomic Epidemiology (CGE) platform with spaTyper software v1.0 for spa type identification (31), and SCCmecFinder v1.2 for *SCCmec* typing and subtyping (32). ABRicate v1.0.1 was combined with different databases, such as ResFinder and Bacterial Antimicrobial Resistance Reference Gene NCBI, to detect antibiotic resistance genes, the possible induction of resistance, and the detection of virulence factor genes with the Virulence Factor Data Base (33). For detecting the genes encoding virulence factors, we also analyzed the genomic assemblies in the CGE platform in combination with the VirulenceFinder software v2.0.3. We used the NG-CHM Builder: Cluster Matrix platform from the University of Texas (https://build.ngchm.net/NGCHM-web-builder/) to analyze the virulence factors profiles detected.

Finally, a phylogenetic analysis was performed from the assembled bacterial genomes using Roary v3.13.0, IQ-Tree v1.5.5, and FigTree v.1.4.4 (http://tree.bio.ed.ac.uk/software/figtree/), in order to establish the core genome of the clinical isolates analyzed (34, 35). The pan-genomic analysis was carried out using only the SNP alignment with SNP sites v2.5.1, and a Newick phylogenetic tree was derived to depict the relationships between each strain (36). The output files generated by Roary software were visualized in the online tool Microreact at https://docs.microreact.org/ (37), generating the Newick-based tree with the genotypes and demographic characteristics of the isolates.

In the pan-genomic analysis and to provide regional context to this study, the public genomic data from another 15 MRSA isolates that caused invasive infections in humans (isolated from the bloodstream) and collected in reference health centers of five countries in the southern cone of South America during 2019 by the StaphNet-SA Consortium were also included . A representative clone from each participating country (Argentina, Bolivia, Brazil, Paraguay, and Uruguay) and from the main MRSA clonal complexes (CC30, CC5, and CC8) identified by Di Gregorio et al. (38) were included in the study (Table S2) under the project accession number PRJEB37318.

We also included data from the following public reference strains in the pan-genomic analysis: MRSA 252–MRSA CC30-ST36-IIA (NC_002952.2), TCH60–MRSA CC30-ST30-IV (NC_017342.1), NCTC8325–MSSA CC8-ST8 (NC_007795.1), N315 MRSA CC5-ST5-IIA (NC_002745.2), and USA300 MRSA CC8-ST8-IV (NZ_CP092052.1).

The rationale for this was to obtain a broader perspective on the genomic relationship of the isolates studied and improve the validity of the results obtained.

All the bioinformatics software used in this study was run using default parameters.

## RESULTS

### Phenotypic and molecular characteristics of the MRSA isolates studied

The Microbiology Department biobank currently has 622 isolates of *S. aureus* collected from 2009 to 2013, of which 148 (23.8%) are MRSA, and 39 of them have caused invasive infections in children. The latter were all isolated from a normally sterile body site such as blood, purulent discharge, tissue, or synovial fluid. Initially, antibiotic susceptibility testing, clonal identification, and molecular characterization of the 39 MRSA isolates causing invasive infections were carried out by MLST, spa typing, identification of the *SCCmec cassette,* MLVA, PFGE, and detection of the PVL coding gene (Table S1).

The main MRSA clonal complex causing invasive infections in Paraguayan children that circulated in the period studied (*N* = 39, 2009–2013) were CC30 53.8% (21/39), CC5 38.5% (15/39), and CC8 7.7% (3/39). We determined the resistant phenotypic rates for nine antibiotics in all the MRSA isolates, which were found to be sensitive to vancomycin (39/39), and the resistant levels found were 97.4% (38/39) penicillin, 25.6% (10/39) erythromycin, 20.5% (8/39) clindamycin, 12.8% (5/39) gentamicin, 5.1% (2/39) for tetracycline, rifampin, and ciprofloxacin, and 2.6% (1/39) trimethoprim-sulfamethoxazole.

The most important MRSA clonal complex, due to its abundance, was CC30 (21/39), mainly constituted by the clone CC30-ST30-MRSA-IV-t019 (20/21), pulsotype A and subtypes (13/21). All of them (21/21) were MLVA profile 1, resistant to penicillin, sensitive to gentamicin, trimethoprim-sulfamethoxazole, and ciprofloxacin. Also, most of them were sensitive to tetracycline and rifampin (20/21), with resistance to macrolides found in four isolates (4/21), and 11/21 carried the PVL gene. The isolates of the CC30 were collected in all the hospitals participating in the study, in different periods of the time included in the study.

The second most frequent and the more variable clonal complex was the CC5 (15/39), which was composed of two sequence types (ST5 and ST100) and six different spa types (t311, t002, t149, t7078, t1791, and t1062).

The main clone that represents CC5 is CC5-ST5-MRSA-IV-t311-PVL+ (8/15), MLVA profile 2, pulsotype B, and subtypes (7/8), resistant to penicillin, sensitive to gentamicin, tetracycline, rifampin, and ciprofloxacin. Also, they were mostly sensitive to trimethoprim-sulfamethoxazole (7/8), with resistance to macrolides found in 2/8 isolates.

The other clones of the CC5 (7/15) were very variable, including one MRSA isolate, CC5-ST5-MRSA-I-t149, pulsotype B, MLVA profile 2, and resistant to penicillin, gentamicin, macrolides, and ciprofloxacin.

The molecular analysis also revealed 3/39 MRSA isolates from the CC8-ST8/72-IV, resistant to penicillin, sensitive to trimethoprim-sulfamethoxazole, clindamycin, rifampin, and ciprofloxacin.

Using this molecular information and due to the budget limitations, we carefully selected the most representative isolates of the main MRSA clones causing invasive infections in Paraguayan children that circulated in the study period for WGS. The MRSA isolates selected for WGS are indicated in gray color in Table S1. Clinical, phenotypic, and genotypic characteristics of MRSA selected are shown in Table 1. All MRSA isolates (10/10) came from patients of both sexes under 15 years of age. All these MRSA isolates (10/10) were resistant to penicillin and sensitive to vancomycin, gentamicin, ciprofloxacin, trimethoprim-sulfamethoxazole and have the *mecA* gene in the *staphylococcal cassette SCCmec* IV.

**TABLE 1:** Features of MRSA that cause invasive infection in Paraguayan children (*n* = 10)^*a*^

Sample ID	Collection date	Sample	Patient disease	CC	MLST	Spa type	MLVA	SCCmec	PFGE	*lukF*/*S-PV*
SGP11	Dec 2009	Blood	Pneumonia	30	30	t019	1	IV	A	+
SIP29	Jan 2010	Blood	Osteomyelitis	30	30	t019	1	IV	A	+
SGP29	Feb 2010	Blood	Sepsis	5	5	t311	2	IV	B	+
SGP63	Nov 2011	Blood	Sepsis	8	8	t11770	4	IV	C1	+
SGP102	Jan 2012	Blood	Sepsis	30	30	t019	1	IV	A	+
SCM71	Feb 2012	Purulent discharge	Sepsis	5	100	t002	3	IV	B6	−
SCM77	Apr 2012	Purulent discharge	Sepsis	5	5	t311	2	IV	B	+
SHN80	Nov 2012	Blood	Sepsis	30	30	t021	1	IV	A4	−
GIP4	Aug 2013	Blood	Osteomyelitis	8	8	t400	5	IV	E	+
GIP64	Dec 2013	Blood	Pneumonia	30	30	t019	1	IV	A	+

^*a*^ ID, identification; CC, clonal complex; and *lukF/S-PV*, Panton-Valentine leukocidin gene.

### Resistome analysis

All the isolates that were subject to WGS carried the *mecA*-resistant gene, which confers resistance to methicillin. The susceptibility profile, the *SCCmec IV* subtyping, and the resistome to the most important antibiotics associated with the treatments of staphylococcal infections are shown in Table 2.

**TABLE 2:** Susceptibility profile and resistome of MRSA that cause invasive infection in Paraguayan children (*n* = 10)^*a*^^,^^*b*^^,^^*c*^^,^^*f*^

Sample ID	CC-ST-spa type	FOX/β-lactams^*d*^	*Cassette SCCmec*	PEN	VAN^*e*^	GEN	Macrolides genotypic/ERY,CLY	Macrolides resistance phenotype	TET	CLO	FOS	RIF
SGP11	CC30-ST30-t019	*mecA*/R	IVc (2B)	*blaZ*/R	*−*/S	−/S	*ermC*/R, R	iMLS	*tet (38*)/S	*cat(pC221*)/ND	*fos-B-Saur*/ND	−/S
SIP29	CC30-ST30-t019	*mecA*/R	IVc (2B)	*blaZ*/R	−/S	−/S	−/S, S	–	*tet (38*)/S	*cat(pC221*)/ND	*fos-B-Saur*/ND	−/S
SGP29	CC5-ST5-t311	*mecA*/R	IVa (2B)	*blaZ*/R	−/S	−/S	*ermC*/R, R	iMLS	*tet (38*)/S	*cat(pC221*)/ND	*fos-B-Saur*/ND	−/S
SGP63	CC8-ST8-t11770	*mecA*/R	IVc (2B)	*blaZ*/R	−/S	−/S	−/S, S	–	*tet (38*)/R	*cat(pC221*)/ND	*fos-B-Saur*/ND	*rphC*/S
SGP102	CC30-ST30-t019	*mecA*/R	IVc	*blaZ*/R	−/S	−/S	−/S, S	–	*tet (38*)/S	*cat(pC221*)/ND	*fos-B-Saur*/ND	*rphC* /S
SCM71	CC5-ST100-t002	*mecA*/R	IVc (2B)	*blaZ*/R	−/S	*aac(6′)-aph(2″*) /S	−/S, S	–	*tet (38*)/S	−/ND	*fos-B-Saur*/ND	*rphC*/R
SCM77	CC5-ST5-t311	*mecA*/R	IVa (2B)	*blaZ*/R	−/S	−/S	−/S, S	–	*tet (38*)/S	−/ND	*fos-B-Saur*/ND	−/S
SHN80	CC30-ST30-t021	*mecA*/R	IVa	*blaZ*/R	−/S	*ant (9)-la*/S	*ermA/*R, R	iMLS	*tet (38*)/R	−/ND	*fos-B-Saur*/ND	−/S
GIP4	CC8-ST8-t400	*mecA*/R	IVc (2B)	*blaZ*/R	−/S	−/S	−/S, S	*–*	*tet (38*)/S	*cat(pC221*)/ND	*fos-B-Saur*/ND	*rphC* /S
GIP64	CC30-ST30-t019	*mecA*/R	IVc (2B)	*blaZ*/R	−/S	−/S	−/S, S	*–*	*tet (38*)/S	−/ND	*fos-B-Saur*/ND	−/S

^*a*^ Table S3 contains the precise information about each resistance gene identified in the MRSA isolates (*n* = 10), with the identity/coverage percentage, position in the reference/contig, data base, and accession number.

^*b*^ Database: Resfinder + National Center for Biotechnology Information (NCBI) + Argannot.

^*c*^ Other antibiotic resistance genes looked at by WGS but not detected in the MRSA isolates analyzed were mupirocin, fusidic acid, ciprofloxacin, trimethoprim-sulfamethoxazole, quinupristin + dalfopristin, spiramycin, linezolid, telithromycim, teicoplanin, and tiamulin.

^*d*^ β-Lactams tested phenotypically: cefoxitin and oxacillin.

^*e*^ Susceptibility to vancomycin was determined by E-test.

^*f*^ (–), gene not detected by WGS; S, phenotypically sensitive; R, phenotypically resistant; ND, no data; ID, identification; ST, sequence type; FOX, cefoxitin; PEN, penicillin; VAN, vancomycin; GEN, gentamicin; ERY, erythromycin; CLY, clindamycin; TET, tetracycline; CLO, chloramphenicol; FOS, fosfomycin; and RIF, rifampin.

For all resistance genes identified, the sequence identity percentages ranged between 83.9% and 100% and had a breath of coverage between 81.7% and 100%, except the *cat(pC221*) gene for the SGP63 isolate, with a 100% identity and 60.6% coverage (Table S3).

The antibiotic susceptibility phenotype and genotype identified were 100% concordant for beta-lactams, glycopeptides, and macrolides (Table 2). Although we do not have phenotypic data on susceptibility to teicoplanin, tigecycline, ceftaroline, linezolid, fosfomycin, and chloramphenicol in the MRSA isolates studied, we did not find genetic evidence of antimicrobial determinants associated with the resistance to teicoplanin, tigecycline, ceftaroline, and linezolid in the WGS analysis. In this regard, the underlying databases used for genome annotation included the possibility to assess genes that confer resistance to the following antimicrobial classes: beta-lactam, aminoglycoside, glycopeptide, quinolone, amphenicol, macrolide, lincosamide, tetracycline, streptogramin a and b, fosfomycin, pseudomonic acid, steroid antibacterial, folate pathway antagonist, oxazolidinone, pleuromotulin, peroxide, quaternary ammonium compound, aminocyclitol, polymyxin, nitroimidazole, aldehyde, and rifampin.

Despite the phenotypic sensitivity to vancomycin, we did not find any of the gene variants associated with glycopeptide resistance in the studied MRSA isolates (note that the *Van* genes that could be assessed were *VanA, VanH, VanX, VanHAX, VanE, VanXY, VanHBX, VanC1XY, VanC2XY, VanC3XY, VanC4XY, VanHDX, VanHFX, VanEXY, VanGXY, VanG2XY, VanLXY, VanHMX, VanNXY, VanHOX,* and *VanXmur*). Table S4 includes all the point mutations associated with resistance genes found in the isolates.

### Virulome analysis

**Fig 1:**
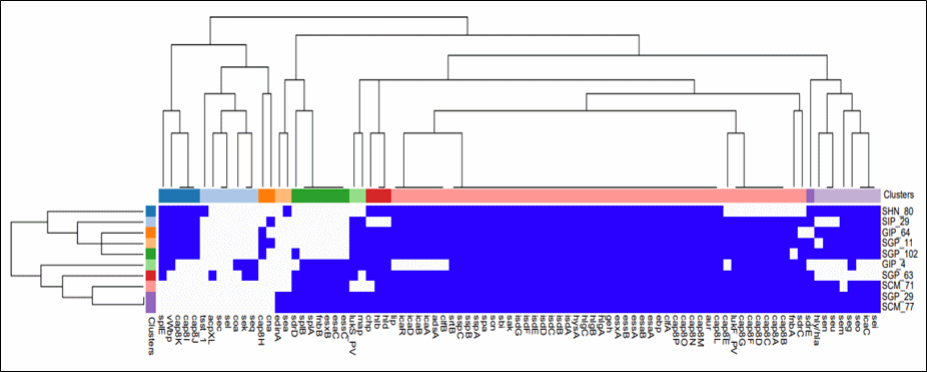
Virulence factors’ heat map and dendrogram from the *de novo* assembly of MRSA genomes from Paraguayan children (*n* = 10). Blue boxes indicate the presence of each one of the virulence factors encoding the genes analyzed (*n* = 87). The isolates were clustered hierarchically based on their virulence factor profile using Euclidean metric distance with complete linkage clustering in both rows and columns, thus providing two dendrograms. The top dendrogram (top) clusters virulence factor clustered according to their frequency in the isolates: the most frequent in the middle and the least frequent in the extremes. The left dendrogram (left) clustered the isolates in terms of their similarity in virulence profile; within the same cluster are SIP29, GIP64, SGP11, and SGP102, all CC30-ST30-t019-IVc and SHN80 clustering further away corresponding to CC30-ST30-t021-IVa. A separate cluster was composed of SCM77 and SGP29 (both corresponding to CC5-ST5-t311-IV), SCM71 (CC5-ST100-t002-IVc), SGP63 (CC8-ST8-t11770-IVc), and GIP4 (CC8-ST8-t400-IVc).

The 87 virulence factor genes detected are shown in Fig. 1. The toxin and virulence gene contents were diverse and correlated with the typing characteristics (Fig. 1). The isolates were clustered based on their virulence factor profile, providing two dendrograms. According to the frequency of virulence factors in the isolates and the similarity in their virulence profile, SIP29, GIP64, SGP11, and SGP102 (CC30-ST30-t019-IVc) clustered together, while SHN80 (CC30-ST30-t021-IVa) located aside. In another cluster, SCM77 and SGP29 (both CC5-ST5-t311-IVc/a) were grouped as identical alongside SCM 71 (CC5-ST100-t002-IVc); SGP63 and GIP4, both CC8-ST8–IVc, clustered separately.

Considering how the virulome results cluster the MRSA isolates by clonal complex (Fig. 1), we conclude that CC30-ST30-t019-IV is characterized by genes that encode proteins involved in biofilm formation, coagulation and adherence, enterotoxin, pore-forming exotoxins, evasins, and few others proteins with antiphagocytic activity. The analysis of the CC5-ST5-t311-IV virulome revealed the presence of genes encoding enterotoxin A (*seA*), PVL (*lukF/S-PV*), and superantigens (SAgs) encoded in the EGC group (G, I, M, N, O, and U), and the more complete antiphagocytic protein profile, as a determinant of cytotoxic strain.

The CC8-ST8-lVc clones (t11770 and t400) carry the complete serine protease system (*splA/B/E*), as well as the PVL and the enterotoxins K and Q, characteristic of CC8. Both of them carried the complete arginine catabolic mobile element (ACME), associated with an arginine deiminase (*arcAa* and *arcA2*), spermidine N (1)-acetyltransferase (*speG*), putative ATPase copper exporters (*copB1* and *copB2*), and a putative lipoprotein (*lipo*). We also assessed the copper and mercury resistance (COMER) mobile element, associated with an abortive phage infection system (abi-α) and two main gene clusters, the *mer* operon composed of the *merR/A*/*B* genes and the *cop* operon composed of the *copB/L/mco* genes. However, we did not find abi-α or the *mer* operon in any of the MRSA genomes.

All the isolates studied carry the genes associated with biofilm formation. Only the CC5-ST5-t311-IV-PVL+ carried the *edinA* gene, encoding the epidermal cell differentiation inhibitor.

All isolates carried aureolysin (*aur*), gamma-hemolysin (*hlg*) A, B, and C components, iron-regulated surface determinant protein (*isd*) A, B, C, E, F, and G components, alpha-hemolysin (*hly/hla*), staphylokinase precursor (*sak*), cell surface elastin (*ebp*), and clumping factor A fibrinogen-binding protein (*clfA*). In addition, all of them carry the IgG- and IgA-binding protein (*spa*), complement inhibitor SCIN (*scn*), glycerol ester hydrolase (*geh*), the protein secretion system components (*es*) EsxA, EsaA/B, and EssA/B, and the type 8 capsular polysaccharide synthesis components (*cap8*) L/M/N/O/P, with antiphagocytic functions. The serine protease (*sspA*), staphopain, cysteine proteinase (*sspB*), staphostatin B (*sspC*), and NPQTN-specific sortase B (*srtB*) were also detected in all isolates. The differential presence of the virulence genes detected by WGS is shown in Table 3 for a better understanding and classification according to their functionality. None of the sequenced isolates carried enterotoxins (*se*) B, C, D, H, L, or the exfoliative toxins (*et*) A/B.

**TABLE 3:** Virulome of the sequenced MRSA that causes invasive infection in Paraguayan children (*n* = 10)^*a*^

Protein type/function	Antiphagocytic					Pore-forming exotoxins		Adherence		Exoenzyme		Toxins	Evasins
ID sample CC-ST-spatype	CP	SSP	SAPM	CI	ECDI	PVL	Hemolysin	BFG	SF	CF	TGD	SA	SP
SGP11 CC30-ST30-t019	*cap8A/B/C/D/E/* *F/G/H/I/J/K*	–	*adsA*	*Chp*	*–*	*lukF-PV,* *lukS-PV*	*hlb, hld*	*icaA, icaB, icaC, icaD,* *icaR*	*sdrC, sdrE, cna,* *fnbA, map*	*clfB, vWbp*	*lip*	*seg*, *sem, sen, seo,* *seu*	*splE*
SIP29 CC30-ST30-t019	*cap8A/B/C/D/E/* *F/G/I/J/K*	–	*adsA*	–	–	*lukF-PV,* *lukS-PV*	–	*icaA, icaB, icaC, icaD,* *icaR*	*sdrC, sdrE, cna, fnbA, map*	*clfB, vWbp*	*lip*	*seg, sem, seo*	*splE*
SGP29 CC5-ST5-t311	*cap8A/B/C/D/E/* *F/G*	*esaC, essC, esxB*	*adsA*	*Chp*	*edinA*	*lukF-PV,* *lukS-PV*	*hlb, hld, hly/hla*	*icaA, icaB, icaC, icaD,* *icaR*	*sdrC, sdrD, sdrE, fnbA,* *fnbB, map*	*clfB*	*lip*	*sea, seg, sei, sem,* *sen, seo, seu*	*splA, splB*
SGP63 CC8-ST8-t11770	*cap8A/B/C/D/E/* *F/G*	*esaC, essC, esxB*	*adsA*	*Chp*	–	*lukF-PV,* *lukS-PV*	*hlb, hld*	*icaA*, *icaB, icaD,* *icaR*	*sdrC, sdrD, sdrE, fnbA,* *fnbB*	*clfB*	*lip*	*sek, seq*	*splA, splB, splE*
SGP102 CC30-ST30-t019	*cap8A/B/C/D/E/* *F/G/H/I/J/K*	–	*adsA*	*Chp*	–	*lukF-PV,* *lukS-PV*	*hlb, hld, hly/hla*	*icaA, icaB, icaC, icaD,* *icaR*	*sdrC, sdrD, sdrE, map*	*clfB, vWbp*	*lip*	*seg, sei, sem,* *sen, seo, seu*	*splE*
SCM71 CC5-ST100-t002	*cap8A/B/C/D/E/* *F/G*	*esaC, essC, esxB*	*adsA*	*–*	*–*	*lukF-PV*	*hlb, hld, hly/hla*	*icaA, icaB, icaC, icaD,* *icaR*	*sdrC, sdrD, sdrE, fnbA,* *fnbB*	*clfB*	*lip*	*seg, sei, sen,* *seo, seu*	*splA, splB*
SCM77 CC5-ST5-t311	*cap8A/B/C/D/E/* *F/G*	*esaC, essC, esxB*	*adsA*	*Chp*	*edinA*	*lukF-PV,* *lukS-PV*	*hlb, hld, hly/hla*	*icaA, icaB, icaC, icaD,* *icaR*	*sdrC, sdrD, sdrE, fnbA,* *fnbB, map*	*clfB*	*lip*	*sea, seg, sei, sem,* *sen, seo, seu*	*splA, splB*
SHN80 CC30-ST30-t021	*cap8I/J/K*	*–*	*adsA*	*Chp*	*–*	*–*	*hlb, hld, hly/hla*	*icaA, icaB, icaC, icaD,* *icaR*	*sdrE*	*clfB, vWbp*	*lip,*	*sea, seg,sei, sem,* *sen,* *seo, seu, tsst-1*	*splE*
GIP4 CC8-ST8-t400	*cap8A/B/C/D/F/* *G*	*esaC, essC, esxB*	*–*	*Chp*	*–*	*lukF-PV,* *lukS-PV*	*hlb, hld*	*–*	*sdrC, fnbA,* *fnbB, map*	*coa, vWbp*	*–*	*seg, sek, seq*	*splA, splB, splE*
GIP64 CC30-ST30-t019	*cap8A/B/C/D/E/* *F/G/H/I/J/K*	*–*	*adsA*	*Chp*	*–*	*lukF-PV,* *lukS-PV*	*hlb, hld, hly/hla*	*icaA, icaB, icaC, icaD,* *icaR*	*fnbA*	*vWbp*	*lip*	*seg, sei, sem,* *sen, seo, seu*	*splE*

^*a*^ (–), gene not detected by WGS; ID, identification; CC, clonal complex; ST, sequence type; CP, capsular polysaccharide; SSP, secretion system protein; SAPM, synthases of anti-phagocytic mediators; CI, chemotaxis-inhibiting; ECDI, epidermal cell differentiation inhibitor; PVL, Panton-Valentine leucocidin; BFG, biofilm formation genes; SF, surface proteins; CF, coagulation factors; TGD, triacylglycerol degradation; SA, superantigens; and SP, serine proteases.

### Phylogenomic analysis

The sequence type and spa type detected by WGS were fully concordant with the results obtained by PCR (Table 1).

**Fig 2:**
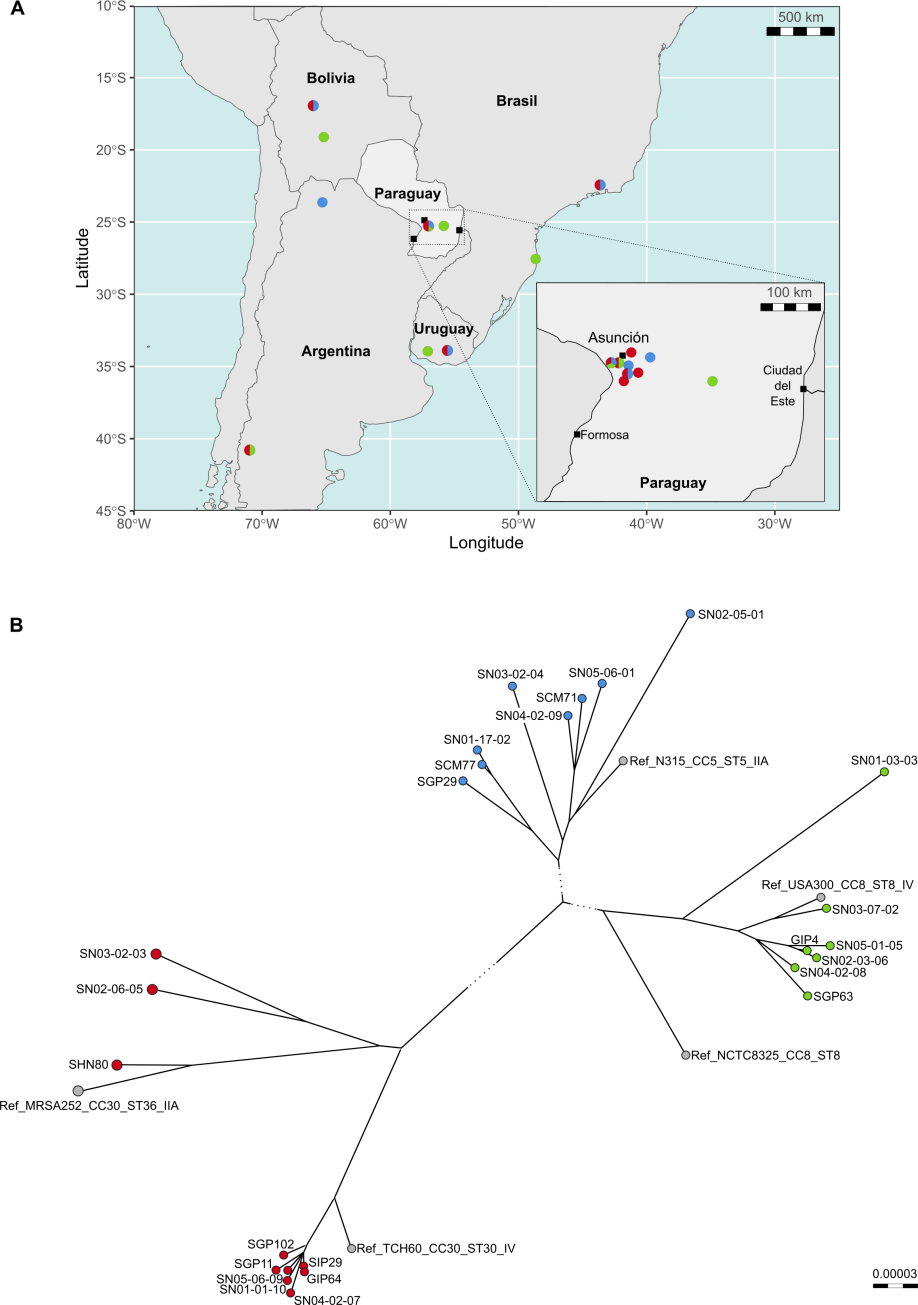
Phylogeny, molecular, and demographic characteristics of MRSA that cause invasive infection in Paraguayan children along with reference data sets and with representative sequences from South America. (**A**) Geographical distribution of samples collected in Paraguay (inset), together with selected samples from Di Gregorio et al. (38). (**B**) Midpoint-rooted phylogenetic tree inferred after aligning the 10 genomes of the study (CC30: SGP11, SIP29, SGP102, GIP64, and SHN80; CC5: SGP29, SCM77, and SCM71; CC8: SGP63 and GIP4), 15 samples from Di Gregorio et al. (38), and 5 reference sequences (shown in gray). Nodes are colored by clonal complex (CC) of strain as well as indicated in panel A: CC30 in red, CC5 in blue, and CC8 in green. Intercluster branches (shown as dotted lines) are not at scale to improve the intracluster resolution of relationships. Data are available at https://microreact.org/project/dzzEoQaWvAJzdHTxt75hdj-primary-methicillin-resistant-staphylococcus-aureus-clones-causing-invasive-infections-in-paraguayan-children.

Whole genome phylogenetic analysis, including 15 selected samples from Di Gregorio et al. (38) and 5 *S*. *aureus* reference sequences, clustered the isolates into three distinct clades: CC30-ST30 (containing SGP11, SIP29, SGP102, GIP64, and SHN80), CC5-ST5/ST100 (containing SGP29, SCM71, and SCM77), and CC8-ST8 (containing SGP63 and GIP4). The geographical distribution and phylogenetic tree are shown in Fig. 2.

## DISCUSSION

Despite the burden of MRSA infection in Paraguay, this constitutes the first WGS analysis of MRSA isolates from this country. MRSA clones represent a significant health concern worldwide. There is a clear need to better understand their genomic structure, transmission dynamics, and evolution in a geographic region over time (1). In this study, using conventional molecular methods, we characterized MRSA isolates that caused invasive infection between 2009 and 2013 in Paraguayan children. They were collected in four high-complexity reference hospital centers in the country, which provide health coverage to a large percentage of the population (39). All the genetically linked MRSA isolates were recovered from diverse clinical sources, patients, and hospitals at broad gap periods, reducing the probability of them being from an outbreak.

We identified three clonal complexes circulating, the most important being CC30, followed by CC5 and CC8. These data represent a change with respect to the first report of Paraguayan MRSA clones prior to that dates (40), where the predominant clonal complex was CC5 (85%), mainly CC5-ST5-I, followed by CC8 (17%), mainly CC8-ST239-IIIA collected in 2005 in one capital city hospital, and reinforce the results reported by Rodríguez et al. (14) in 2020, where CC30 was already reported as the main clonal complex in CA-MRSA isolates (80%), followed by CC5 (16%) and other minority clonal complexes such as CC8 (2%).

The most representative clone for its abundance is the CC30-ST30-IV-t019 (21/39), similar to other reports in Argentina and South America, which indicate that the most prevalent and geographically disseminated MRSA is the ARG-4: CC30-ST30-IVc-t019, characterized by specific mobile genetic elements and chromosomal mutations that might have contributed to its virulence and success (38, 41).

In this study and due to economic limitations, in the WGS analysis, we only included the most representative MRSA clones causing invasive infections circulating in Paraguayan children between 2009 and 2013, such as the CC30-ST30-IV (*n* = 5), CC5-ST5-IV (*n* = 3), and CC8-ST8-IV (*n* = 2), to evaluate their genetic diversity, virulence factor repertoire, and the antimicrobial resistance determinants.

The phylogenomic analysis revealed three major and different clonal complexes (CC30, CC5, and CC8), each composed of clones closely related to each other and to the public and reference genomes included in the study.

The CC30 proves to be a successful clone, strongly installed and disseminated throughout the country in this study and documented since 2009 in previous studies by our research group (14) and up to 2019 by recent studies carried out by the StaphNET-SA Consortium (38).

The CC30 genomes from this phylogenomic study (*n* = 5) demonstrate that they were closely related to the CC30 public genomes TCH60 (CC30-ST30-IV) and MRSA252 (CC30-ST36-IIA) and to MRSA genomes from Argentina, Bolivia, Brazil, Paraguay, and Uruguay, collected from the bloodstream in 2019 by the StaphNET-SA Consortium (38), providing stronger evidence that these clones, particularly the CC30-ST30-IV, are installed in the main hospital centers of Paraguay and in the region, and that it is not seasonal since it appears in any month of the year.

The presence of a new clone of the CC30-ST30-IV lineage, related to the most frequently reported CC30-ST30-IV-t019 but with spa type t021, shows that this clone begins to differentiate in the *spA* gene sequences, in this case, in three repetitions by insertion and deletion. This phenomenon generally appears in chronic or repeated infections, indicating that clone CC30-ST30-t019 is probably beginning to change, evolving to other spa types (15).

The main MRSA clones circulating in Paraguay show differences in their virulence profiles. CC30-ST30-t019-IV is characterized by having the largest number and diversity of genes encoding capsular polysaccharides, which express proteins with antiphagocytic activity and inhibit the interaction between C3b, immunoglobulin, and receptors. This clonal complex (CC30) has gained genes that encode the coagulation factor protein (*vWbp*) and superantigens (SAgs) encoded in the EGC cluster, all of which are involved in aggravating the infectious process (42–44). The superantigens (SAgs) are currently considered the most prevalent staphylococcal toxins among clinical and colonizing isolates. They are present in 50%–70% of nasal carriers, and their expression may be crucial for the development and aggravation of some infections, such as respiratory and endocarditis (42, 45). It has been shown in animal models that the expression of the EGC cluster and *tsst-1* genes together contributes to an increase in mortality and/or a more rapid and complicated progression of the infection, generally accompanied by lethal complications, such as heart failure and cerebrovascular accidents. Even so, clinical infections progress in the presence of EGC cluster toxins, even without the systemic or local effects of TSST-1. Therefore, it is postulated that the EGC cluster toxins are also responsible for the worsening of the infections, probably on a smaller scale than TSST-1 (46).

This well-known virulent strain caused skin lesions, sepsis, and pneumonia in children and young adults in hospitals and the community. In recent descriptive studies, this clone has been associated with an increased risk of infective endocarditis (47) and persistent bacteremia due to *S. aureus* (48).

The CC5-ST5-t311-IV clones studied here carried genes encoding some specific virulence factors as a signature of highly toxic strains, involved in CA-MRSA infections. This is even more significant because these isolates were from invasive infections. The SCM_71 strain, CC5-ST100-t002-IV and resistant to rifampin, known as the pediatric clone, is highly related to CC5-ST5-t311-IV and differs from it by a point mutation in the *aroE* gene (substitution of the aroE4 allele, characteristic of ST5, by the aroE65 characteristic of ST100), as well as by the insertion of a repeat r17 in the fifth position of the *spA* gene that differentiates it from t311 and converts it into spa type t002 (49). The CC5-ST100-t002-IV clone could be a pathway on the CC5-ST5-t311-IV evolution, allowing its adaptation to the sanitary conditions of the region (50).

The CC8-ST8-lVc-PVL+ clones carried the serine protease system complex (*splA/B/E*), a digestive system extracellular with a role in the pathogenesis of MRSA, PVL, and the enterotoxins K and Q, characteristics of the CC8 complex, among others, all of which contributing to their virulence profile (1). These strains carried the complete ACME, denoting that our isolates collected between 2009 and 2013 were closely related to the CC8-ST8-IVc-PVL+ (USA300-NAE-ACME+), identified as the major cause of CA-MRSA infections in North America, which have lately been increasingly reported worldwide (8). This situation has changed, and the USA300-NAE-ACME+ was displaced in Paraguay and in the region, based on the latest report by Di Gregorio et al. (38), in which USA300LV or USA300-SAE-COMER+ are currently found in the region and in Paraguay.

In the present study, almost all the MRSA analyzed (38/39) carried the *cassette SCCmec IV,* which is smaller than the others, has a simpler genetic makeup, and generally carries only the methicillin resistance genes (*mecA*) (1). This is probably the reason why our isolates still showed high phenotypic sensitivity levels to drugs used in serious MRSA infectious diseases, such as glycopeptides, ceftaroline, and linezolid, and do not present multiple drug resistance (as defined by Magiorakos; )^51^). However, the increasing levels of resistance to macrolides and lincosamides detected in this study, compared to previous reports in our country (14, 52), which exceed the cut-off point of 10% considered a reasonable limit for the use of clindamycin in the empirical treatment of mild or moderate skin and soft tissue infection caused by MRSA (53), highlight the possibilities of therapeutic failure when using it and may directly affect the empirical scheme that must be taken into account in daily pediatric practice.

Regarding the resistome analysis, most of the MRSA isolates showed phenotypic and genotypic concordance with the antibiotic susceptibility results. Similar findings were reported in other studies that showed a concordance in 76%–100% of isolates (54, 55). Our results showed differences between genotype and phenotype in isolates that carried resistant genes to tetracycline, rifampin, and gentamicin. This could be due to mutations in these resistance genes or defects in their expression that should be further explored experimentally in the future.

On the other hand, we assessed all genes and point mutations associated with the VISA and hVISA phenotypes of resistance in each of our samples (56, 57). However, the isolates lack any of the specific genes or mutations reported to date. Instead, we found other point mutations in these genes that have not been associated with the hVISA phenotype. This reconciles with the VSSA phenotype found in the MRSA strains analyzed.

We also obtained data for other antimicrobials that are not routinely tested in the laboratory, such as fosfomycin and chloramphenicol, detecting their potential resistance in 10/10 and 4/10 of MRSA isolates, respectively. Fosfomycin and chloramphenicol are not used to treat staphylococcal infections in Paraguay and Argentina. This is the reason why it was not included in the antibiotic panel (41).

Regarding the virulome of the isolates, we identified the co-existence of multiple virulence factor groups, such as the coding pore-forming exotoxins, cell wall-anchored surface proteins, exoenzyme, toxins, evasins, and some antiphagocytic functions proteins, showing that they all have a vital function for survival, reproduction, colonization, and bacterial spread, which could explain why they are part of the *S. aureus* core genome (49, 58, 59).

CC30-ST30-IV and CC8-ST8-IV clones win the serine protease *splE* genes, encoding evasins with possible cooperative and complementary activities with the other *spl* proteases (lost by CC30), and apparently constitute an extracellular digestive system with a role in the pathogenesis of *S. aureus* (46). In contrast, the loss of the secretion system proteins used by bacteria to interact and manipulate their environments is significant for adhesion and permanence in the host cell (60), as well as the epidermal cell differentiation inhibitor (*edinA*), which is involved in the bacterial dissemination process and hindering complement-mediated phagocytosis (61). This lack of genes could give these lineages (CC30 and CC8) better efficiency for propagation due to their smaller size and the lower fitness costs associated with carrying fewer genes, favoring those involved in the evasion of the immune response, infection expansion, and propagation process, and being an explanation of it displacing the other lineages (1).

We identified the potential ability for biofilm formation in almost all the MRSA isolates analyzed, except for the CC8-ST8-t400-IV clone. The biofilm is another crucial factor contributing to staphylococcal infections, implicated in various persistent human microbial infectious diseases, allowing them to evade multiple clearance mechanisms, such as antimicrobials and the host immune system leading to treatment failure and recurrent/chronic infections. MRSA biofilm and virulence factor production are closely linked since the primary biofilm regulator, the accessory gene regulator *agr,* is also vital for expressing numerous virulence factors. Therefore, many biofilm-related virulence factors have been the target of research on *S. aureus* therapeutics (62). Biofilm formation, especially in chronic infections, is crucial since it affects the choice of strategies for their elimination, such as surgical removal, given the ineffectiveness of traditional antibiotic therapies (63, 64).

In conclusion, the use of WGS in the present study added value to the classic isolate identification and molecular typing protocols. It offered precious and precise genomic data about the most prevalent MRSA clones identified in this study. Multiple virulence and resistance genes were identified for the first time in this study, indicative of the complex virulence profiles of MRSA that are circulating in Paraguay. This critical qualitative leap opened a wide range of new possibilities for future projects and trials to improve the existing knowledge on the epidemiology of MRSA circulating in Paraguay.

## Data Availability

All assemblies from this study are available at the NCBI Sequence Read Archive (BioProject accession number PRJNA830493.

## SUPPLEMENTAL MATERIAL

Supplementary Table S1 spectrum.03012-23-s0001.xlsx MRSA isolates that caused infections in Paraguayan children, N=39 (2009-2013).

Supplementary Table S2 spectrum.03012-23-s0002.xlsx MRSA public genomes from five countries of the southern cone of South America during 2019 by the StaphNet-SA Consortium (DiGregorio et al., 2023).

Supplementary Table S3 spectrum.03012-23-s0003.xlsx Resistance genes identified in the MRSA analyzed by NGS.

Supplementary Table S4 spectrum.03012-23-s0004.xlsx Point mutations associated with resistance genes found in the MRSA isolates analyzed.

ASM does not own the copyrights to Supplemental Material that may be linked to, or accessed through, an article. The authors have granted ASM a non-exclusive, world-wide license to publish the Supplemental Material files. Please contact the corresponding author directly for reuse.
